# Docosahexaenoic acid, but not eicosapentaenoic acid, improves septic shock-induced arterial dysfunction in rats

**DOI:** 10.1371/journal.pone.0189658

**Published:** 2017-12-20

**Authors:** Alexandra Boivin, Mélanie Burban, Raphaël Clere-Jehl, Pierrick Le Borgne, Hamid Merdji, Cyril Auger, Valérie Schini-Kerth, Ferhat Meziani, Julie Helms

**Affiliations:** 1 Université de Strasbourg (UNISTRA), Faculté de Médecine, Hôpitaux universitaires de Strasbourg, service de réanimation, nouvel hôpital civil, Strasbourg, France; 2 INSERM (French National Institute of Health and Medical Research), UMR 1260, Regenerative Nanomedicine (RNM), FMTS, Strasbourg, France; 3 Service d'accueil des Urgences, Hôpital de Hautepierre, CHU de Strasbourg, 1, Strasbourg, France; 4 Université de Strasbourg, INSERM, EFS Grand Est, BPPS UMR-S 949, FMTS, Strasbourg, France; Max Delbruck Centrum fur Molekulare Medizin Berlin Buch, GERMANY

## Abstract

**Introduction:**

Long chain n-3 fatty acid supplementation may modulate septic shock-induced host response to pathogen-induced sepsis. The composition of lipid emulsions for parenteral nutrition however remains a real challenge in intensive care, depending on their fatty acid content. Because they have not been assessed yet, we aimed at determining the respective effects of eicosapentaenoic acid (EPA) and docosahexaenoic acid (DHA) during septic shock-induced vascular dysfunction.

**Methods:**

In a peritonitis-induced septic shock model, rats were infused with EPA, DHA, an EPA/DHA mixture or 5% dextrose (D5) during 22 hours. From H18, rats were resuscitated and monitored during 4 hours. At H22, plasma, aorta and mesenteric resistance arteries were collected to perform ex vivo experiments.

**Results:**

We have shown that septic rats needed an active resuscitation with fluid challenge and norepinephrine treatment, while SHAM rats did not. In septic rats, norepinephrine requirements were significantly decreased in DHA and EPA/DHA groups (10.6±12.0 and 3.7±8.0 μg/kg/min respectively versus 17.4±19.3 μg/kg/min in D5 group, p<0.05) and DHA infusion significantly improved contractile response to phenylephrine through nitric oxide pathway inhibition. DHA moreover significantly reduced vascular oxidative stress and nitric oxide production, phosphorylated IκB expression and vasodilative prostaglandin production. DHA also significantly decreased polyunsaturated fatty acid pro-inflammatory mediators and significantly increased several anti-inflammatory metabolites.

**Conclusions:**

DHA infusion in septic rats improved hemodynamic dysfunction through decreased vascular oxidative stress and inflammation, while EPA infusion did not have beneficial effects.

## Introduction

Over the past decades, the composition of lipid emulsions for parenteral nutrition has emerged as a real challenge in intensive care. Indeed, lipid emulsions may differently interfere with inflammation and immune pathways, depending on their fatty acid content [1–3]. N-3 polyunsaturated fatty acid (PUFA) supplementation, including eicosapentaenoic acid (EPA) and docosahexaenoic acid (DHA), was thus proposed as a potential beneficial adjunctive therapy [4,5]. Yet, in critically ill patients, lipid emulsions for parenteral nutrition containing n-3 PUFA are used daily in intensive care units (ICUs) if enteral nutrition is contraindicated [1]. Septic shock is one of the first causes of ICU mortality, and is characterized by severe hypotension with a profound vasodilation and vascular hyporesponsiveness to vasoconstrictors [6,7]. Although n-3 PUFA supplementation is considered as a promising therapeutic in several inflammatory diseases [4,8,9], including septic shock patients [1], the respective effects of EPA and DHA in vascular dysfunction of septic shock have never been established yet. Whether only their combination or both of their individual effects are beneficial has not been determined either.

Marine n-3 PUFAs may be beneficial in septic shock patients by decreasing the deregulated hyper-inflammatory response. Indeed, n-3 PUFAs have anti-inflammatory effects by modulating cell membrane and lipid raft compositions, thus notably interfering with innate and adaptive immune cell differentiation and functions [10–12]. They are also responsible for the inhibition of inflammatory gene expression, the activation of anti-inflammatory transcription factor, and the modulation of transcription by binding to peroxisome proliferator-activated receptors (PPARs) [1]. Their effective potential benefits however remain contradictory and largely unexplained. Thus, while Heller et al. [13] showed a significant mortality reduction in critically ill patients treated with parenteral fish oil emulsion, there was no benefit in septic patients [14]. Both Barbosa et al. [15] and Gultekin et al. [16] supported these results in smaller cohorts of sepsis or septic shock patients.

EPA and DHA could have synergistic effects, as assessed by recent data. Zhang et al. [17] thus showed that EPA and/or DHA supplementation reduced the inflammatory response in a rat ischemia-reperfusion model and this effect is more important when EPA and DHA are combined. Interestingly, Allaire et al. [18] showed that DHA is more effective than EPA in modulating inflammation markers and blood lipids in humans at risk of cardiovascular disease.

The purpose of our experimental study was therefore to determine in an original way the dissociated and combined effects of the EPA and DHA parenteral administration in a rat peritonitis-induced septic shock model and assess the mechanisms involved in their hemodynamic and vascular effects.

## Material and methods

This study was performed with the approval of the Strasbourg Regional Committee of Ethics in Animal Experimentation (CREMEAS, AL/69/76/02/13).

Male Wistar rats weighing 400±20 g were randomly allocated to 8 groups (n = 10 rats/group): SHAM-D5, SHAM-EPA, SHAM-DHA, SHAM-EPA/DHA, CLP-D5, CLP-EPA, CLP-DHA and CLP-EPA/DHA.

### Septic shock model and infusion with n-3 PUFAs

Rats underwent cecal ligation and puncture (CLP) as previously described [19]. During surgical procedures, rats were anesthetized with isoflurane 1–2% (Baxter S.A.S, Maurepas, France) and analgesia was performed with subcutaneous buprenorphine (Sogeval, Laval, Fance; 0.1 mg/kg of body weight;). A subcutaneous injection of 0.1 mL lidocaine 1% (AstraZeneca, Rueil-Malmaison, France) was performed before skin incision. Under aseptic conditions, a 3-cm midline laparotomy was performed to allow exposure, ligation and puncture of the caecum with a 21-gauge needle. A small amount of feces was extruded, the caecum returned into the peritoneal cavity and the laparotomy closed. SHAM rats underwent a midline laparotomy and cecal exposure without further manipulation. All rats received a subcutaneous injection of 0.9% NaCl after the surgical procedure (30 ml/kg of body weight).

The right femoral vein was then catheterized, and the catheter was tunneled between the ears. Purified EPA and DHA (> 95% purity) from Pivotal Therapeutics, Inc. (Woodbridge, ON, Canada) were emulsified in 10% DMSO (Sigma Aldrich, Saint-Quentin Fallavier, France) (EPA:DHA 1:1). The randomly allocated 2% emulsion (EPA or DHA or EPA:DHA 1:1) was blindly infused at identical rates during 22 hours (0.15 g/kg/day as recommended in humans). The control groups (SHAM-D5 and CLP-D5) were infused with 5% dextrose (D5) at identical rates (S1 Fig). It has to be noted that all the experiments have also been performed using only DMSO as a control at the same concentration as the lipid mediator. DMSO did not affect any parameter considered.

### Hemodynamic monitoring and resuscitation

The CLP-rats developed septic shock within the 16–20 hours after CLP. Septic shock was considered established on the basis of clinical criteria (lethargy, piloerection and glassy eyes) and confirmed if MAP was below 90 mmHg and plasma lactate level elevated (> 2 mmol/L). After 18 hours, rats were anesthetized with an intraperitoneal injection of pentobarbital sodium (60 mg/kg), tracheotomised and mechanically ventilated (Series Small animal Ventilator, SAR-830/P; CWE, Ardmore, PA). Analgesia was ensured as described above.

The left femoral artery was cannulated and used to measure mean arterial pressure (MAP) and heart rate (HR), and collect blood samples. The homolateral femoral vein was cannulated for fluid maintenance and drug infusion. In septic rats, fluid resuscitation was performed by bolus of 0.9% NaCl (500 μL/10 min. when needed) and norepinephrine infused to target a MAP above 90 mmHg. DHA, EPA, EPA/DHA or dextrose infusion was continued at the same rate during the 4-hour resuscitation.

After a 4-hour resuscitation (S1 Fig), blood samples were collected and rats were sacrificed by intravenous pentothal injection (100 mg/Kg). The presence of caecum necrosis and intra-abdominal pus in CLP-rats and their absence in SHAM rats were systematically checked. Aorta was collected for electron paramagnetic resonance measurements. Mesenteric resistance arteries (MRAs) were collected for vascular reactivity study and Western blotting analysis.

### Vascular reactivity of isolated mesenteric arteries

Second-order rat mesenteric arterial rings were mounted on a wire-myograph (DMT, Aarhus, DK). Two stainless steel wires (25 μm in diameter) were inserted into the lumen of the arteries and respectively fixed to a force transducer and a micrometer. The experiments were performed at 37°C in a physiological salt solution (Krebs) with the following composition (in mM): 119 NaCl, 4.7 KCl, 25 NaHCO_3_, 1.17 MgSO_4_/7H_2_O, 2.5 CaCl_2_, 1.18 KH_2_PO_4_ and 11 glucose, continuously bubbled with 95% O_2_ and 5% CO_2_. After an equilibration period (at least 20 min.) under optimal passive tension, arteries viability was tested using a potassium rich physiological salt solution (80 mM) and phenylephrine (Phe, 10 μM) (Sigma-Aldrich, Saint Quentin Fallavier, France) was used to test the maximal contractile capacity of the vessels. Endothelial function was assessed by acetylcholine (ACh) ability to induce relaxation (80–100% of precontracted vessels). Cumulative concentration-response curves to Phe, vasoconstrictor agonist (0.1 nM to 100 μM) were performed. After 20-min. washout period, to study endothelium-dependent relaxation, mesenteric arterial rings with functional endothelium were precontracted with Phe (10 μM) and then exposed to increasing incremental concentrations of ACh (0.1 nM to 100 μM) (Sigma-Aldrich, Saint Quentin Fallavier, France). Cumulative concentration-response to Phe and ACh were performed in the presence or absence of indomethacin (10 μM) (Sigma-Aldrich, Saint Quentin Fallavier, France), nitro-L-arginine (L-NA, 30 mM) (Sigma-Aldrich, Saint Quentin Fallavier, France) for 20 min.: the first vessel with no inhibitor, the second with nitro-L-arginine and indomethacin.

### Electron paramagnetic resonance (EPR) analysis

Nitric Oxide Spin Trapping (NO). Detection of NO production was performed using a previously described technique with Fe^2+^ diethyldithiocarbamate (DETC) as a spin trap. Determination of NO formation was assessed by electron spin resonance spectroscopy after formation of [Fe(II)NO(DETC)_2_], the paramagnetic DETC complex with NO, in aorta samples. Briefly, aorta samples were washed twice with Hank’s balanced salt solution buffered with 10 mM 4-(2-hydroxyethyl)-1-piperazineethanesulfonic acid and then incubated in a Hank’s balanced salt solution-4-(2-hydroxyethyl)-1-piperazineethanesulfonic acid solution in the presence of bovine serum albumin (20.5 mg/mL), 3 mM CaCl_2_, 0.8 mM L-arginine for 15 minutes at 37°C. Spin trap chemicals FeSO_4_ (2.25 mg) and NaDETC (3.6 mg) were separately dissolved under nitrogen gas bubbling in 10 mL volumes of ice-cold Hank’s balanced salt solution-4-(2-hydroxyethyl)-1-piperazineethanesulfonic acid buffer. The solutions were rapidly mixed to obtain a pale yellow-brown opalescent colloid [Fe(II)5DETC)2] solution (final concentration 0.4 mM), which was used immediately to incubate samples for 45 min. at 37°C. After incubation, the spin trap was removed and samples were put in physiological salt solution and frozen in calibrated tubes in liquid nitrogen for electronic paramagnetic resonance measurements. NO measurement was performed on a tabletop x-band spectrometer Miniscope (Magnettech, MS200, Berlin, Germany). Recordings were performed at 77°K, using a Dewar flask. Instrument settings were 10 mW of microwave power, 1 mT of amplitude modulation, 100 kHz of modulation frequency, 60s of sweep time and five number of scans.

Superoxide Anion Spin-Trapping (O_2_^.-^). Aorta samples were incubated in deferoxamine-chelated Krebs-4-(2-hydroxyethyl)-1-piperazineethanesulfonic acid solution containing 1 hydroxy-3methoxycarbonyl 2,2,5,5-tetramethylpyrrolidin (500 μM; Noxygen, Elzach, Germany), deferoxamine (25 μM, Sigma Aldrich), and DETC (Sigma Aldrich; 5 μM) under constant temperature (37°C) for 1 hour. The reaction was stopped by placing the samples in ice, subsequently frozen in liquid nitrogen for EPR spectroscopy analysis.

Values were expressed in signal amplitude/mg weight of dried tissue (Amplitude/Wd).

### Western immunoblotting

Tissues from harvested MRAs, aortas and hearts were homogenized in lysis buffer (10 mM Tris-HCl, containing 1% sodium dodecyl sulfate, 1 mM sodium orthovanadate, 2.5 mg/l leupeptin and 5 mg/l aprotinin, pH 7.4) using an Ultrasonic Processor (Bioblock Scientific, Illkirch, France). Lysates were centrifuged at 4°C. Twenty micrograms protein of MRA lysates were then loaded onto 8% SDS-polyacrylamide gels and electro-blotted on a nitrocellulose membrane (Amersham Biosciences, Buckinghamshire, UK). Blots were probed by an overnight incubation (4°C) with a mouse anti-inducible nitric oxide synthase (iNOS) antibody (BD Biosciences, Le Pont de Claix, France), a polyclonal rabbit nuclear factor NF-κB p65 antibody (Abcam, Cambridge, UK), a mouse anti-human phosphorylated (ser32/36)-IκB alpha (pIκB) antibody (Euromedex, Souffelweyersheim, France), a monoclonal rabbit cyclo-oxygenase 1 (COX-1) (Abcam, Cambridge, UK), a polyclonal rabbit cyclo-oxygenase 2 (COX-2) antibody (Abcam, Cambridge, UK) and a rabbit anti-heme-oxygenase-1 (HO-1) polyclonal antibody (OSA-200, Stressgen Bioreagents). Membranes were washed and incubated for one hour at room temperature with a secondary anti-mouse or anti-rabbit peroxidase-conjugated IgG (Promega, Madison, WI, USA). The immunoreactive proteins were visualized with enhanced chemiluminescence (ECL Plus Western Blotting Detection Reagents, Amersham Biosciences), and the membranes were probed again with a mouse anti-β-actin antibody (Sigma-Aldrich, Saint Quentin Fallavier, France) for densitometric quantification and normalization to β-actin expression. Band intensities were quantified with Quantity One Sofware (ImageQuant LAS 4000 series, GE Healthcare Europe, Velizy-Villacoublay, France).

### Quantification of pro-resolving EPA and DHA metabolites

To 100 μL of plasma 300 μL of cold methanol and 5 μL of internal standard (Deuterium labeled compounds) were added. After centrifugation at 900 g for 15 min at 4°C, supernatants were transferred into 2 mL 96-well deep plates and diluted in H_2_O to 2 mL. Samples were then submitted to solid phase extraction (SPE) using OASIS HLB 96-well plate (30 mg/well, Waters) pretreated with MeOH (1mL) and equilibrated with 10% MeOH (1 mL). After sample application, extraction plate was washed with 10% MeOH (1 mL). After drying under aspiration, lipids mediators were eluted with 1 mL of MeOH. Prior to LC-MS/MS analysis, samples were evaporated under nitrogen gas and reconstituted in 10 μL on MeOH.

LC-MS/MS analyses of eicosanoids were performed as described [20]. Briefly, lipid mediators were separated on a ZorBAX SB-C18 column (2.1 mm, 50 mm, 1.8 μm) (Agilent Technologies) using Agilent 1290 Infinity HPLC system (Technologies) coupled to an ESI-triple quadruple G6460 mass spectrometer (Agilent Technologies). Data were acquired in Multiple Reaction Monitoring (MRM) mode with optimized conditions (ion optics and collision energy). Peak detection, integration and quantitative analysis were done using Mass Hunter Quantitative analysis software (Agilent Technologies) based on calibration lines built with commercially available eicosanoids standards (Cayman Chemicals).

### Quantification of thromboxane A_2_ and prostacyclin I_2_

Thromboxane B_2_ (TXB_2_), derived from rapid non-enzymatic hydrolysis of TXA_2_, and prostacyclin I_2_, which is rapidly non-enzymatically hydrated to a stable product, 6-keto PGF1α, were measured in plasma using the respective immunoassay kit (ELISA) according to manufacturer’s instructions (Cayman Chemical Company, Ann Arbor, MI, USA).

### Statistical analysis

Data obtained during measurements of arterial pressure and heart rate were compared using a nonparametric Wilcoxon signed rank test. Two-way analysis of variance for repeated measurements was used for comparison of vascular reactivity (data were tested for homogeneity of variance by Levene's statistics), and nonparametric Kruskal-Wallis test or Mann-Whitney test were used for comparison Western blotting, NO, superoxide, immunostaining signal measurements and metabolite quantification between the four groups. When a significant difference was found between groups, subsequent post hoc tests were performed. All these tests were performed with the Statview version 5.0 software (SAS Institute, Cary, NC). P < 0.05 was considered statistically significant. The effects of Ach were quantified as the percentage of relaxation in preparations previously contracted with Phe. All values are presented as mean ± SD for n experiments. n represents the number of animals.

## Results

### DHA infusion decreased norepinephrine needs during septic shock in rats

Compared to SHAM rats, septic rats were characterized by a significant hypotension (MAP) refractory to fluid challenge at the end of the experiment (H22) (SHAM-D5: 142±15 mmHg versus 83±22, 74±28, 91±24 and 110±23 mmHg in CLP-D5, CLP-EPA, CLP-DHA and CLP-EPA/DHA respectively, p<0.05) and required an active resuscitation with significant norepinephrine infusion (SHAM-D5: 0±0 μg/kg/min versus 17.4±19.3, 24.7±28.2, 10.6±12.7 and 3.7±7.9 μg/kg/min in CLP-D5, CLP-EPA, CLP-DHA and CLP-EPA/DHA respectively, p<0.05). Compared to CLP-D5 and CLP-EPA rats, cumulative norepinephrine doses were significantly lower in DHA and EPA/DHA groups (p<0.05). Compared to SHAM rats, plasma lactate level in septic rats was significantly increased (SHAM-D5: 1.2±0.4 mM versus 3.4±1.6, 3.7±2.0, 3.2±1.0 and 2.8±0.8 mM in CLP-D5, CLP-EPA, CLP-DHA and CLP-EPA/DHA respectively, p<0.05).

### DHA infusion improved septic shock-induced arterial dysfunction

**Fig 1:**
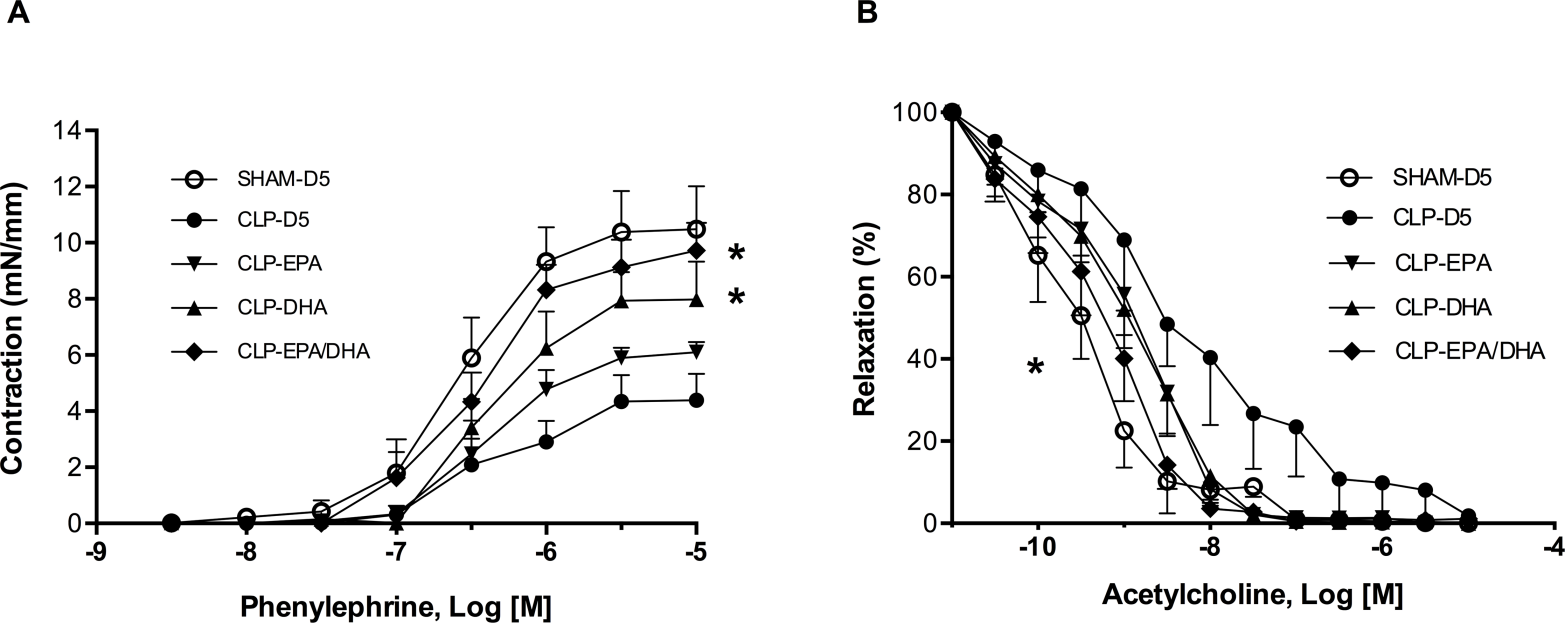
DHA infusion improved septic shock-induced arterial dysfunction. A. Mesenteric resistance artery contractile response to phenylephrine (Phe); * p<0.05 vs. CLP-D5. B. Mesenteric resistance artery relaxation to acetylcholine (ACh), * p<0.05: half maximal effective concentration (EC_50_) CLP-EPA/DHA vs. EC_50_ CLP-D5. CLP: cecal ligation and puncture, n = 10 rats/group.

Ex vivo MRAs of CLP-D5 rats showed a significant hyporeactivity to phenylephrine (Table 1). Both DHA and EPA/DHA, but not EPA alone, improved ex vivo contractile response to phenylephrine compared to CLP-D5 group (p<0.05) (Fig 1A). Nitric oxide synthase and COXs pharmacological inhibition significantly restored the vascular reactivity to phenylephrine in CLP-D5 rats; without inhibitors, DHA and EPA/DHA, but not EPA alone, exerted the same effect in term of contractility levels (Table 1).

**Table 1:** Vascular reactivity of mesenteric resistance arteries.

Mesenteric resistance arteries Contraction (mN/mm)				
Products	SHAM-D5 CLP-D5	SHAM-EPA CLP-EPA	SHAM-DHA CLP-DHA	SHAM-EPA/DHA CLP-EPA/DHA
Phe	10.2 ± 1.5 4.3 ± 0.9 *	9.0 ± 1.6 5.8 ± 0.6 *	10.2 ± 1.2 8.5 ± 1.6	11.4 ± 1.0 9.7 ± 1.0
Phe+LNA	10.3 ± 1.1 6.4 ± 1.6 ^#^	8.3 ± 0.9 6.8 ± 0.9	9.4 ± 1.4 7.9 ± 1.3	9.5 ± 1.6 8.6 ± 0.8
Phe+Indo	11.4 ± 1.7 8.7 ± 0.6 ^£^	10.9 ± 1.0 8.8 ± 0.3 ^£^	11.8 ± 0.8 9.3 ± 1.4	11.3 ± 1.5 9.4 ± 0.8
Phe+LNA+Indo	10.7 ± 2.2 9.4 ± 2.4 ^£^	10.3 ± 2.0 8.5 ± 1.2 ^£^	12.1 ± 3.3 8.9 ± 2.0	11.4 ± 2.1 10.3 ± 2.8

Values are expressed as mean ± SD; n = 7 rats/group. LNA: L-nitro-arginine; Phe: phenylephrine; Indo: indomethacin.

* p<0.05 vs. vessels harvested from SHAM rats.

^#^ p<0.05 vs. vessels harvested from CLP rats in presence of Phe+LNA.

^£^ p<0.05 vs. vessels harvested from CLP rats in presence of Phe+Indo.

Compared to basal contraction, NO synthase inhibition did not significantly enhance the contractile response in treated septic rats, whatever the group considered (Table 1). COX inhibition alone, as well as the association of NOS and COX inhibition, significantly enhanced contractile response in CLP-EPA rats, but not in in CLP-EPA/DHA rats (Table 1).

The sensitivity to acetylcholine was significantly decreased in CLP group compared to SHAM rats (half maximal effective concentration EC_50_: 115 nM vs. 20 nM respectively, p<0.05). The treatment by EPA-DHA (EC_50_: 91nM) but not EPA or DHA alone (EC_50_: 91 nM and 97 nM respectively) significantly affected the MRAs from CLP group (p<0.05) (Fig 1B).

### DHA infusion reduced septic shock-induced vascular inflammation, oxidative stress and nitric oxide production

**Fig 2:**
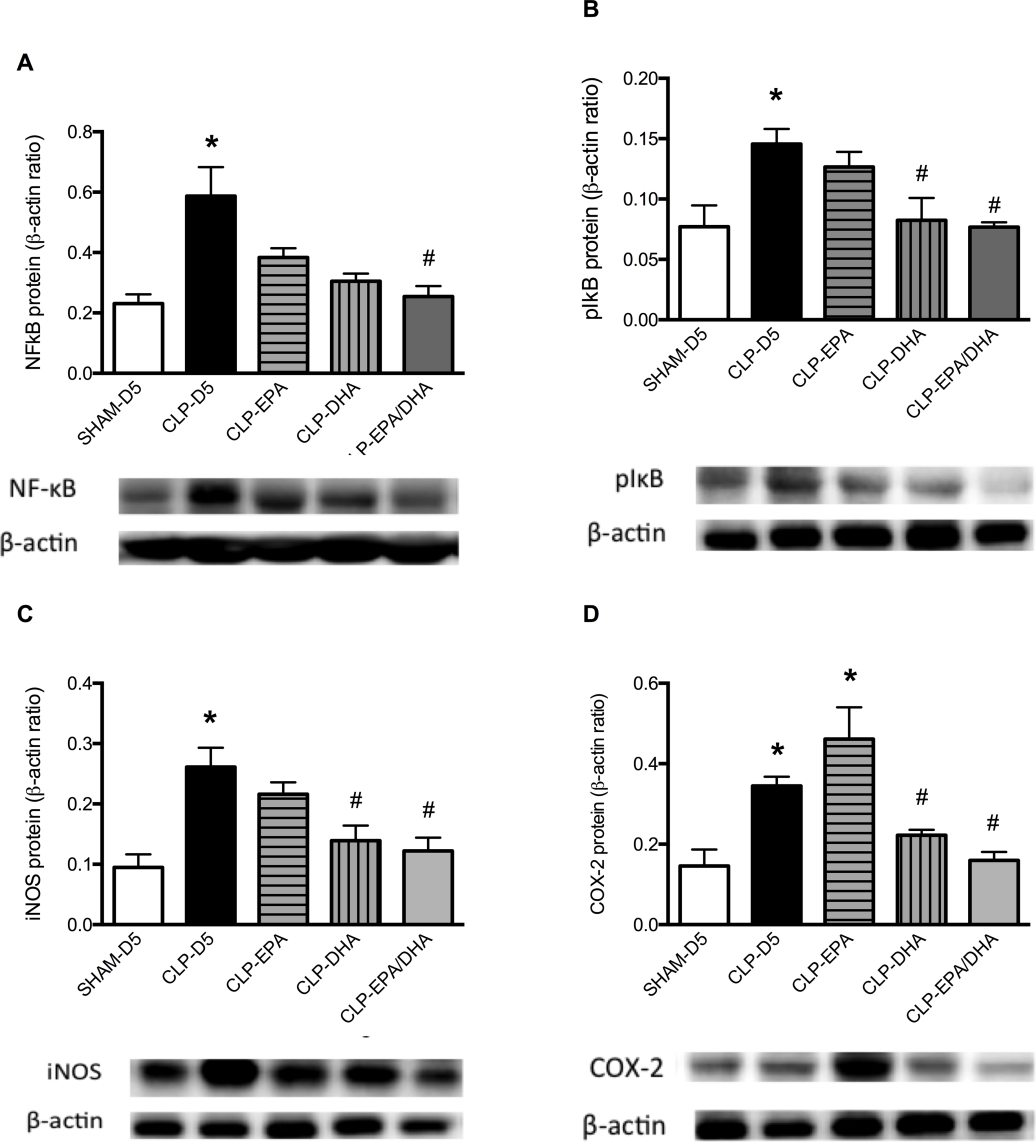
DHA infusion reduced septic shock-induced inflammatory proteins. The NF-κB p65, pIκB, iNOS, COX-2 expressions relative to β-actin content were evaluated by Western immunoblotting in mesenteric arteries. n = 8 rats/group, * p<0.05 vs. SHAM-D5, # p<0.05 vs. CLP-D5 group.

NF-κB activation was assessed by IκB phosphorylation, resulting in free NF-κB nuclear translocation and pro-inflammatory gene transcription. In the MRAs, NF-κB p65 expression was significantly decreased in CLP-EPA/DHA group compared to CLP-D5 group (Fig 2A). In MRAs, pIκB expression was significantly increased in CLP-D5 and CLP-EPA rats compared to SHAM-D5 rats, and decreased in CLP-DHA and CLP-EPA/DHA rats compared to CLP-D5 rats (Fig 2B).

**Fig 3:**
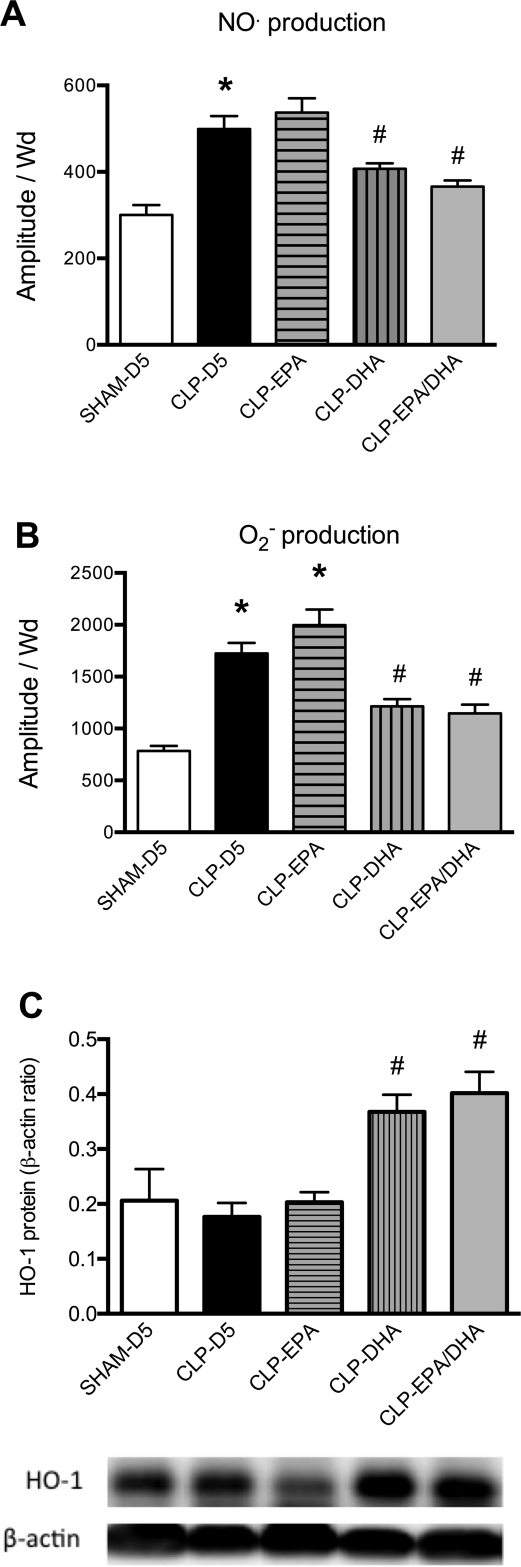
DHA infusion reduced septic shock-induced vascular oxidative stress and nitric oxide production. Nitric oxide and superoxide anion productions were measured by electronic paramagnetic resonance in the aorta and heme oxygenase-1 (HO-1) expression relative to ß-actin content evaluated by Western immunoblotting in mesenteric arteries. n = 8 rats/group, * p<0.05 vs. SHAM-D5, # p<0.05 vs. CLP-D5 group.

Inducible NOS expression was significantly increased in MRAs from septic rats compared to SHAM rats and decreased in MRAs from CLP-DHA and CLP-EPA/DHA groups, compared to CLP-D5 and CLP-EPA groups (Fig 2C). Cyclo-oxygenase-1 expression did not significantly differ between the groups (not shown). Compared to SHAM rats, COX-2 expression was significantly increased in MRAs from septic rats and decreased in MRAs from CLP-DHA and CLP-EPA/DHA groups, but not in CLP-EPA group (Fig 2D). Compared to SHAM group, NO and O_2_^-^ productions were significantly increased in the aorta from CLP-D5 rats. DHA and EPA/DHA infusions significantly decreased this production. Nitric oxide and O_2_^-^ productions were not significantly different between CLP-D5 and CLP-EPA (Fig 3A and 3B). In MRAs, HO-1 expression was significantly increased in MRAs from CLP-DHA and CLP-EPA/DHA rats, compared to MRAs from CLP-D5 and SHAM-D5 rats, and not significantly different between SHAM-D5, CLP-D5 and CLP-EPA groups (Fig 3C).

### EPA and DHA infusions altered anti-inflammatory polyunsaturated fatty acid metabolite productions

Anti-inflammatory polyunsaturated fatty acid metabolites (MaR1: maresin 1; LxA4: lipoxin A4; LxB4: lipoxin B4; PGD2: prostaglandin D2; PGE3: prostaglandin E3; PD1: protectin D1; RvD1: resolvin D1; RvD2: resolvin D2; 14- and 17-HDoHE: 14- and 17-hydroxy-docosahexaenoicacid; 18-HEPE: 18-hydroxyeicosapentaenoic acid) were significantly decreased in the plasma of CLP-D5 rats, compared to SHAM rats (Table 2).

**Table 2:** Polyunsaturated fatty acid metabolites.

Metabolite (pg/mL)	Sham	CLP-G5	CLP-EPA	CLP-DHA	CLP-EPA/DHA
**MaR1**	220517±92875	52764±25071^$^	114916±1632^$^	77746±19797*	155307±52957*^#^
**RvD1**	6382±4127	850±630^$^	0±0^$^	2810±660*^#^	5563±1333*^#^
**RvD2**	6424±0	0±0^$^	9±18^$^	5165±986*^#^	4776±1697*^#^
**17-HDoHE**	610067±850	239426±608^$^	54826±7722	298487±520^#^	568140±1333*^#^
**14-HDoHE**	420568±4984	228827±10724^$^	358659±177709	275781±53559	394933±66795
**PD1**	192341±63452	25258±5086^$^	3200±1281^$^	46041±13179^#^	153589±25438*^#^
**18-HEPE**	33478±7560	18298±4629^$^	41811±25538	15114±2010	42681±7332
**PGE3**	362±56	224±58^$^	393±175	422±77	582±81
**LxA4**	105442±19531	23029±5296^$^	5385±1632^$^	55547±10824*^#^	94682±14648*^#^
**LxB4**	15614±1258	3783±1020^$^	5496±3649	12458±914	15987±1762
**PGD2**	13076±878	5489±728^$^	1057±274^$^	10517±1631*^#^	15704±1277*^#^

Values are expressed as mean ± SD; n = 5 rats/group.

* p<0.05 vs. CLP-G5 rats

^#^ p<0.05 vs. CLP-EPA rat

^$^ p<0.05 vs. SHAM rats

MaR1: maresin 1; RvD1: resolvin D1; RvD2: resolvin D2; 17-HDoHE: 17-hydroxy-docosahexaenoicacid; 14-HDoHE: 14-hydroxy-docosahexaenoicacid; PD1: protectin D1; 18-HEPE: 18-hydroxyeicosapentaenoic acid; PGE3: prostaglandin E3; LxA4: lipoxin A4; LxB4: lipoxin B4; PGD2: prostaglandin D2.

In septic rats, DHA infusion significantly increased most of these anti-inflammatory metabolites (MaR1, RvD1, RvD2, LxA4, and PGD2) compared to CLP-D5 rats, while EPA alone did not (Table 2). In addition, EPA and DHA co-infusion significantly increased anti-inflammatory 17-HDoHE and PD1 compared to CLP-D5 rats (Table 2).

### DHA infusion reduced septic shock-induced vasodilative prostacyclin I_2_ production

**Fig 4:**
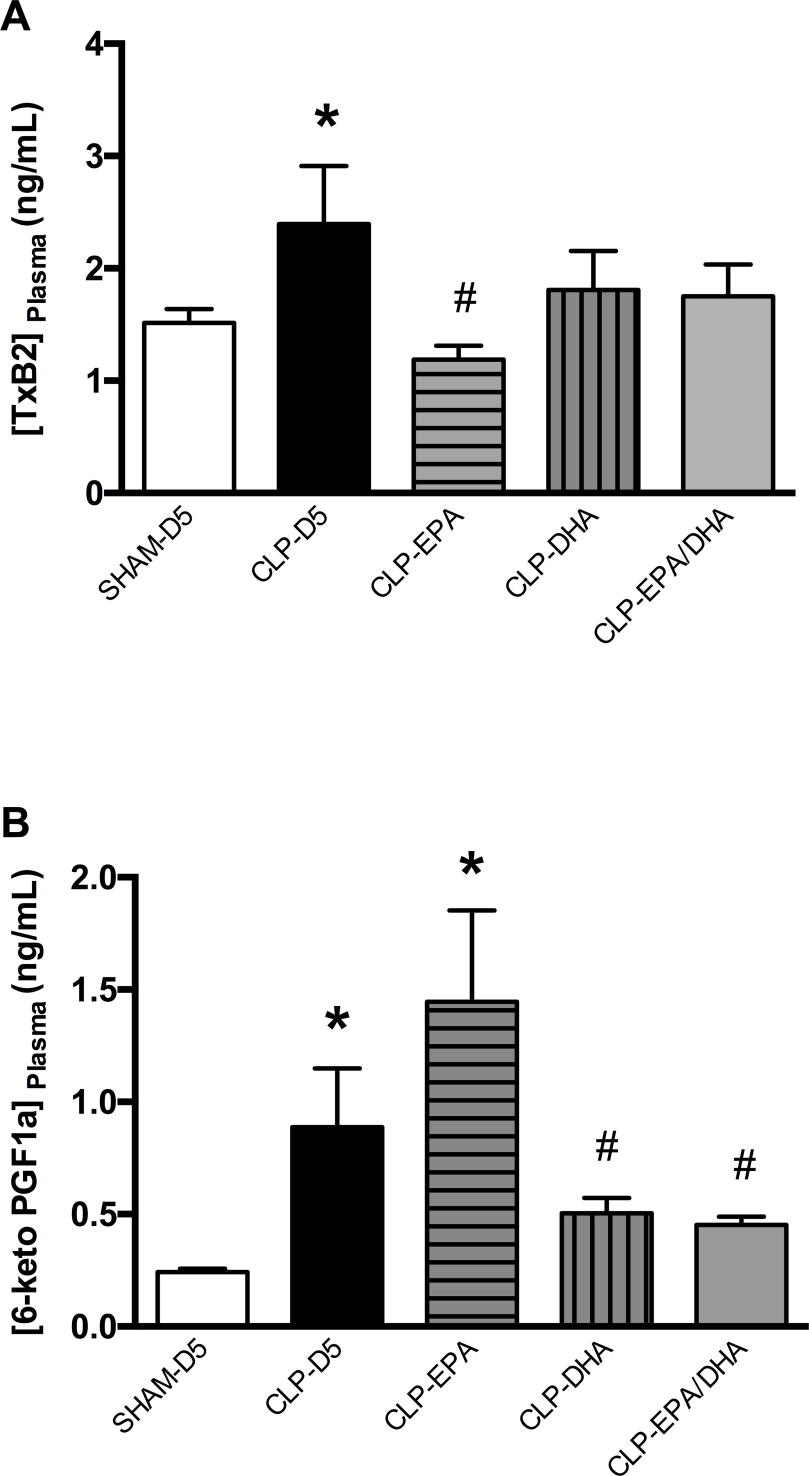
DHA infusion reduced septic shock-induced vasodilative prostacyclin I_2_ production in plasma. A. Plasma levels of thromboxane B2 (TXB_2_) and B. 6-keto PGF1α were measured with immunoassay ELISA kits. n = 8 rats/group, * p<0.05 vs. SHAM-D5, # p<0.05 vs. CLP-D5.

Thromboxane B_2_ level was significantly increased in CLP groups compared to SHAM-D5 group, and decreased in CLP-EPA group compared to CLP-D5 group (Fig 4A).

6-keto PGF1α concentration was increased in MRAs from CLP-D5 and CLP-EPA rats, compared to SHAM-D5, CLP-DHA and CLP-EPA/DHA rats (Fig 4B). The 6-keto PGF1α to TXB_2_ ratio was 0.16±0.03, 0.37±0.13, 0.94±0.24, 0.33±0.11 and 0.31±0.09 in SHAM-D5, CLP-D5, CLP-EPA, CLP-DHA and CLP-EPA/DHA rats respectively, with the ratio being significantly increased in CLP-EPA compared to the other groups (p<0.05).

## Discussion

Although growing evidence suggests that marine n−3 PUFAs may be beneficial in septic shock-induced hyper-inflammatory state, their effective potential benefits remain nonetheless controversial [1,21]. Part of the contradictory results from recent literature may arise from the heterogeneity of the n-3 PUFAs compared. We have thus shown that n-3 PUFAs have differential effects, depending on their nature. For the first time to our knowledge, we describe the respective effects of EPA and DHA supplementations on vascular arterial dysfunction in a septic shock model in rats and the mechanisms involved in these vascular effects. We indeed bring evidence that DHA supplementation decreased vascular inflammation and oxidative stress, improving hemodynamic and arterial dysfunction, while EPA did not.

N-3 PUFA supplementation was shown to decrease organ dysfunction in septic shock patients, ICU and hospital lengths of stay [15,22]. In the present study, we have shown that DHA significantly reduced norepinephrine needs to maintain the MAP target in septic rats, while EPA did not. Barton et al. had already shown that dietary n-3 PUFAs decreased mortality in a rat model of sepsis, but they neither investigated the hemodynamic function, nor the respective effects of EPA and DHA [23]. Consistent with our results, some experimental data suggest that n-3 PUFA supplementation might improve septic shock-induced organ dysfunctions. Indeed, the septic cardiac dysfunction was reduced in rats supplemented with parenteral n-3 PUFAs [24], like in animal models of ischemia-reperfusion induced shock [25,26]. Interestingly, Tunctan et al. [27] have demonstrated that a stable synthetic analog of 20-hydroxyeicosatetraenoic acid (20-HETE), derived from arachidonic acid, prevents vascular hyporeactivity, hypotension and inflammation in endotoxemic rats. Moreover, EPA and DHA may have several effects, notably through the production of different anti-inflammatory mediators, including the E- and D-series resolvins or protectins, depending on the metabolic pathway involved [28]. Interestingly, EPA and DHA could have synergistic effects on inflammation in a model of intestinal ischemia-reperfusion, compared to EPA or DHA alone [17]. In this line, we have shown that only DHA but not EPA could have the same beneficial effect in septic shock-induced vascular dysfunction and these beneficial hemodynamic effects are likely to be in part due to differences in derived vasoactive metabolites after DHA or EPA or EPA+DHA infusion. Indeed, herein DHA infusion significantly decreased the production of vasodilative 6kPGF1 in septic rats and was also associated to the production of some anti-inflammatory D-series resolvins, resulting in the hemodynamic and vascular beneficial effects, through decreased sepsis-induced vasodilation. Consistent with these data, we have shown that supplementing septic rats with DHA or EPA+DHA, but not EPA alone, significantly improved the contractile response and endothelial function of resistance arteries, through nitric oxide pathway inhibition. The result of COX inhibition on isolated arteries permitting to restore the contractile response highlighted the role of derived vasoactive metabolites when septic rats were treated by EPA. Thus, several reports showed that DHA and EPA might have different vascular effects in hypertensive rats and hyperlipidemic patients. Indeed, DHA enhanced vasodilator mechanisms and attenuated constrictor responses, while EPA did not [29]. Thus, it was suggested that DHA alters endothelial function, involving different mechanisms depending on the pathology considered [29]. Moreover, DHA was already reported to exert antioxidative activity, decreasing reactive oxygen species production and NO synthase activity [30–32]. Consistent with these data, we have shown that arterial NO production and oxidative stress were both decreased in arteries from septic rats supplemented with DHA. HO-1 expression is induced by both inflammatory cytokines and oxidative stress [33] during sepsis and other inflammatory diseases. It was shown to improve resistance to oxidative stress and modulate inflammatory response [34–36], through anti-inflammatory and antioxidant properties [37]. In this context, we have shown that HO-1 expression is increased in mesenteric arteries from septic rats treated with DHA and EPA/DHA and may contribute to their anti-inflammatory and antioxidant properties. Furthermore, DHA supplementation decreased inflammation via NF-κB down regulation septic rat arteries and these results are consistent with the literature, which has shown that n-3 PUFAs down regulate NF-κB pathway, inflammatory cytokine and adhesion molecule production, and COX-2 gene transcription [2,38].

Finally, we have shown that EPA supplementation increased the production of vasodilative arachidonic acid-derived prostacyclin I2 in the plasma, which mainly depends on COX-2 activity in the vascular wall [39], consistent with vascular COX-2 increase in this group. Interestingly, the plasma concentration of its biological opposite, thromboxane A_2_, mainly depending on COX-1 and thromboxane synthase, remained unaltered by n-3 PUFAs. The balance between PGI_2_ and TXA_2_ is notably responsible for vascular tone regulation, with PGI_2_ being able to limit the response to TXA_2_ in vivo. PGI_2_/TXA_2_ ratio might be more informative than the absolute level of these prostanoids [39]. Interestingly, the 6-keto PGF1α to TXB_2_ ratio shows a major imbalance with an excessive vasodilative product formation in rats treated with EPA. Mayer et al. [40] demonstrated this beneficial cytokine-shifting effect in septic patients, but once again the EPA and DHA effects were not analyzed separately.

The fact that NO and superoxide was assessed in aorta instead of mesenteric arteries constitutes a limitation of the study. Although both vascular beds depend on different regulatory mechanisms, we have previously shown that sepsis induced the same kind of hyporeactivity to Phe in aorta and small mesenteric arteries [6,41]. Finally, septic shock is a systemic syndrome, in which vascular inflammation, oxidative and nitrosative stresses are ubiquitous, as we showed in our previous studies [42–46].

## Conclusion

The beneficial effects of n-3 PUFAs may be mainly attributable to docosahexaenoic acid, improving vascular dysfunction by decreasing inflammatory mediator production and increasing anti-inflammatory metabolites, and by decreasing oxidative stress and NO production in a rat septic shock model. Thus, purified DHA supplementation might be a promising adjunctive immunonutrition therapy during septic shock, which would deserve further investigations in humans.

## Supporting information

S1 Table Mesenteric resistance arteries: contraction without inhibitor. (PDF)

S2 Table Mesenteric resistance arteries: contraction under L-NA. (PDF)

S3 Table Mesenteric resistance arteries: contraction under Indomethacin. (PDF)

S4 Table Mesenteric resistance arteries: contraction under association of L-NA & Indomethacin. (PDF)

S5 Table Mesenteric resistance arteries: relaxation to Ach without inhibitor. (PDF)

S6 Table Western blots. (PDF)

S7 Table Electronic paramagnetic resonance. (PDF)

S8 Table Plasma levels of thromboxane B2 and 6-keto PGF1α. (PDF)

S1 Fig Protocol design. At H0, rats were randomly allocated to a group and underwent a surgical procedure (either cecal ligation and puncture, CLP, or SHAM) and were infused until H22 by 5% dextrose (D5), purified EPA, DHA or a mixture of EPA and DHA at identical rates. From H18 to H22, rats were anesthetized, ventilated and resuscitated with fluid challenge and norepinephrine to reach the mean arterial pressure (MAP) target over 90 mmHg. At H22, rats were sacrificed. n = 10 rats/group.
(TIF)
