# Supplementary material for: Docosahexaenoic acid, but not eicosapentaenoic acid, improves septic shock-induced arterial dysfunction in rats
Source: PLoS One. 2017 Dec 20;12(12):e0189658. doi: 10.1371/journal.pone.0189658 (PMC5738044; doi:10.1371/journal.pone.0189658)
Supplement: S1 Table — (PDF) [file pone.0189658.s001.pdf]

Table S1

Mesenteric resistance arteries: contraction without inhibitor

	RAT	Phe 10^{-9}	Phe 3.10^{-9}	Phe 10^{-8}	Phe 3.10^{-8}	Phe 10^{-7}
RAT SHAM G	R1	0,00	0,00	0,09	0,00	0,08
	R2	0,01	0,01	-0,04	0,06	0,06
	R3	-0,02	-0,01	0,00	0,00	0,12
	R4	-0,06	-0,06	0,02	0,02	-0,04
	R5	-0,03	0,00	-0,01	-0,03	-0,03
	R6	-0,02	-0,01	-0,04	0,00	0,00
	R7	-0,06	-0,06	0,00	1,64	3,22
	R8	0,06	0,09	0,12	0,06	0,12
RAT SHAM EI	R1	-0,06	0,00	-0,05	-0,03	-0,06
	R2	-0,02	-0,02	0,01	-0,04	-0,06
	R3	0,00	0,00	-0,04	-0,02	1,12
	R4	0,01	0,02	0,01	0,03	0,09
	R5	0,01	0,01	0,01	-0,05	0,00
	R6	0,00	0,00	0,00	0,01	0,15
	R7	0,00	0,00	-0,05	-0,03	0,03
	R8	0,03	0,05	0,04	-0,02	0,06
	R9	-0,06	0,00	0,00	0,00	-0,05
RAT SHAM D	R1	-0,03	0,00	-0,02	-0,05	0,01
	R2	-0,03	0,00	-0,01	-0,03	-0,01
	R3	0,05	0,03	0,03	0,03	0,02
	R4	0,01	0,02	-0,04	0,01	0,08
	R5	0,05	0,05	0,01	0,06	1,02
	R6	0,00	0,19	0,26	0,25	0,29
	R7	0,02	0,00	-0,03	0,06	0,08
	R8	-0,02	-0,02	0,06	0,05	0,18
	R9	0,04	0,05	0,06	0,06	-0,08
RAT SHAM O	R1	-0,08	-0,08	0,06	-0,05	0,09
	R2	0,00	0,00	-0,04	-0,02	0,00
	R3	0,27	0,27	-0,02	0,01	0,01
	R4	0,02	0,02	0,05	-0,05	0,00
	R5	0,00	0,00	0,05	0,05	0,07
	R6	-0,03	-0,03	0,08	0,08	0,32
	R7	0,03	0,03	-0,02	0,08	0,63
RAT SEPSIS G	R1	0,05	0,05	0,08	0,08	0,06
	R2	0,02	0,02	0,00	0,00	0,08
	R3	0,01	0,01	-0,02	0,00	0,01
	R4	0,02	0,02	0,02	0,00	0,04
	R5	0,02	0,02	0,05	-0,01	-0,01
	R6	0,00	0,00	0,00	-0,06	-0,08
RAT SEPSIS E	R1	0,05	0,05	-0,02	0,00	-0,02
	R2	-0,03	-0,03	-0,03	0,00	0,08
	R3	0,11	0,11	0,06	0,12	0,05

	R4	0,03	0,09	0,09	0,18	0,21
	R5	0,00	0,00	0,00	0,00	0,05
	R6	-0,02	-0,02	0,00	-0,06	0,39
	R7	-0,11	-0,11	-0,21	-0,09	-0,09
RAT SEPSIS D	R1	-0,09	-0,09	-0,01	-0,08	-0,12
	R2	-0,08	-0,08	0,06	-0,03	0,04
	R3	-0,03	-0,03	0,01	-0,02	1,19
	R4	-0,11	-0,11	-0,05	-0,08	-0,11
	R5	-0,06	-0,06	-0,06	-0,06	-0,04
	R6	0,15	0,15	0,22	0,17	0,05
	R7	0,02	0,02	0,02	0,00	0,02
RAT SEPSIS C	R1	-0,02	-0,02	0,00	0,02	-0,02
	R2	-0,01	-0,01	-0,03	-0,04	-0,06
	R3	0,03	0,03	0,08	0,01	0,06
	R4	0,09	0,09	0,12	0,15	0,14
	R5	-0,11	-0,11	-0,01	-0,08	0,04
	R6	-0,06	-0,01	0,00	0,00	0,21
	R7	-0,11	-0,11	-0,12	-0,12	-0,14

Phe 3.10^{-7}	Phe 10^{-6}	Phe 3.10^{-6}	Phe 10^{-5}	Phe 3.10^{-5}
1,41	7,96	10,54	11,14	11,15
0,99	12,13	13,44	13,24	13,25
0,25	1,29	6,17	6,60	8,60
0,14	4,91	7,75	7,94	9,89
4,78	6,45	6,65	6,81	8,75
0,17	0,66	6,36	8,25	8,25
5,26	9,94	11,01	11,43	11,44
1,74	3,83	9,76	10,68	10,67
1,46	1,91	7,37	9,45	9,46
0,55	15,61	18,12	18,10	18,10
4,35	7,38	9,97	8,45	8,45
0,08	1,94	3,20	3,25	3,23
0,05	2,14	3,63	5,39	8,39
0,61	5,04	6,60	8,64	8,65
0,03	4,18	5,59	5,90	8,90
0,35	2,72	4,87	6,46	6,46
0,39	5,64	11,04	10,21	10,20
0,64	5,28	7,71	7,51	7,51
0,08	4,96	7,55	8,12	8,13
0,08	6,29	9,16	8,38	8,38
4,42	8,33	8,99	9,26	9,26
2,26	10,74	11,47	11,10	11,11
0,63	11,95	12,84	13,07	13,07
5,16	10,57	11,74	11,81	11,81
2,04	12,85	13,01	13,11	13,10
0,04	5,11	9,07	9,93	9,93
6,22	11,84	13,78	13,34	13,34
0,11	5,69	9,49	9,62	9,61
5,59	9,97	12,38	12,35	12,35
0,03	5,04	9,77	10,18	10,18
0,10	5,49	8,98	11,90	11,88
0,33	8,01	8,51	9,12	9,13
7,31	13,51	13,97	13,60	13,60
0,03	0,61	4,44	6,59	6,59
1,41	5,39	3,63	5,71	5,72
0,01	0,16	1,32	1,98	1,97
0,02	0,88	1,05	1,43	1,44
0,43	0,94	1,67	3,72	3,72
-0,03	4,56	5,33	6,64	6,64
0,08	0,05	2,37	5,57	5,57
0,14	0,39	4,32	6,82	6,82
0,03	1,45	5,25	4,98	4,94

0,21	1,85	2,13	4,15	4,11
0,09	0,11	2,21	5,88	5,88
1,53	6,17	5,28	6,80	6,81
-0,12	4,28	6,61	6,65	6,64
0,02	8,12	10,05	9,91	9,90
0,12	2,53	3,18	5,55	7,54
0,09	3,76	5,99	6,09	6,10
-0,12	0,68	3,57	4,61	6,61
0,00	3,92	7,74	7,97	8,96
0,00	4,76	10,94	11,03	11,09
0,00	0,06	2,25	9,44	9,45
0,15	7,37	10,01	10,29	10,29
-0,06	1,43	5,52	7,44	7,43
0,24	6,43	8,61	8,62	8,60
0,09	4,99	7,99	8,75	8,75
8,52	4,63	11,40	10,11	10,11
0,33	3,34	9,10	10,12	10,14
0,78	1,15	6,39	12,49	12,51
