# Supplementary material for: Docosahexaenoic acid, but not eicosapentaenoic acid, improves septic shock-induced arterial dysfunction in rats
Source: PLoS One. 2017 Dec 20;12(12):e0189658. doi: 10.1371/journal.pone.0189658 (PMC5738044; doi:10.1371/journal.pone.0189658)
Supplement: S2 Table — (PDF) [file pone.0189658.s002.pdf]

Table S2

Mesenteric resistance arteries: contraction under L-NA

	RAT	Phe 10^{-9}	Phe 3.10^{-9}	Phe 10^{-8}	Phe 3.10^{-8}	Phe 10^{-7}
RAT SHAM G	R1	0,00	-0,01	0,02	0,00	0,02
	R2	0,02	-0,02	0,00	-0,06	0,02
	R3	0,01	0,01	0,08	0,06	0,09
	R4	0,00	0,00	0,08	0,02	0,04
	R5	0,00	0,03	0,03	0,04	0,01
	R6	0,00	0,00	0,00	-0,01	-0,03
	R7	-0,05	0,02	-0,06	0,00	0,05
	R8	0,15	0,06	-0,15	-0,20	-0,06
RAT SHAM EI	R1	0,08	0,08	0,09	0,06	0,11
	R2	0,02	0,08	0,00	0,00	0,00
	R3	0,09	0,06	0,11	0,11	0,11
	R4	0,04	0,04	0,06	0,05	-0,01
	R5	-0,12	0,05	0,02	0,15	-0,06
	R6	0,06	0,06	0,08	0,05	0,08
	R7	0,09	0,11	0,11	0,11	0,15
	R8	-0,04	0,05	-0,03	0,00	-0,01
	R9	-0,35	-0,35	-0,26	-0,31	-0,25
RAT SHAM D	R1	-0,06	-0,06	-0,03	-0,06	-0,06
	R2	0,08	0,02	0,06	0,11	0,05
	R3	0,08	0,08	0,06	0,04	0,04
	R4	-0,01	0,02	-0,05	0,00	-0,12
	R5	0,94	-0,11	0,01	-0,01	-0,03
	R6	-0,06	-0,06	-0,08	-0,06	-0,06
	R7	-0,01	-0,01	-0,03	-0,06	0,02
	R8	0,00	0,08	0,05	0,06	0,02
	R9	0,12	0,22	0,20	0,20	0,17
RAT SHAM O	R1	0,08	0,06	0,08	0,09	0,18
	R2	0,02	0,00	-0,08	-0,09	-0,08
	R3	0,02	0,02	0,02	0,02	0,02
	R4	-0,22	0,12	0,21	-0,02	-0,05
	R5	0,00	0,00	0,00	-0,05	-0,06
	R6	-0,02	-0,09	-0,32	-0,46	-0,43
	R7	0,05	0,03	0,06	0,08	0,06
	R8	0,04	0,00	-0,03	0,04	0,02
RAT SEPSIS G	R1	0,01	0,05	0,05	0,06	0,16
	R2	0,06	0,05	0,06	0,09	0,05
	R3	0,00	0,01	-0,02	-0,02	0,08
	R4	-0,03	-0,08	0,00	-0,09	-0,11
	R5	0,06	-0,02	0,08	-0,02	0,06
	R6	0,00	0,06	0,08	0,08	-0,01
RAT SEPSIS E	R1	0,01	0,05	0,05	0,06	0,16

	R2	0,06	0,05	0,06	0,09	0,05
	R3	0,00	0,01	-0,02	-0,02	0,08
	R4	-0,03	-0,08	0,00	-0,09	-0,11
	R5	0,06	-0,02	0,08	-0,02	0,06
RAT SEPSIS D	R1	0,00	0,01	0,00	0,00	-0,02
	R2	0,00	-0,01	0,02	0,00	0,02
	R3	0,08	0,06	0,05	0,14	0,06
	R4	0,19	0,19	0,06	0,19	0,20
	R5	0,02	0,00	0,00	-0,02	0,02
	R6	0,00	0,02	-0,08	-0,01	0,12
RAT SEPSIS C	R1	0,05	0,06	0,11	0,08	0,09
	R2	0,00	0,06	0,03	0,06	0,00
	R3	0,00	-0,01	0,00	0,02	0,05
	R4	-0,01	-0,08	-0,14	-0,11	-0,06
	R5	-0,02	-0,05	-0,15	-0,21	-0,05
	R6	0,17	0,22	0,09	0,06	0,03

Phe 3.10^{-7}	Phe 10^{-6}	Phe 3.10^{-6}	Phe 10^{-5}	Phe 3.10^{-5}
0,05	0,08	2,18	7,02	8,04
0,00	0,60	7,29	14,96	15,39
-0,05	0,14	4,35	8,64	9,05
0,04	0,08	2,24	5,38	5,21
0,06	1,22	7,51	11,61	11,75
-0,03	0,28	6,08	8,06	8,49
0,00	0,06	3,36	7,72	12,24
-0,17	0,25	8,82	13,34	12,35
0,09	2,58	4,52	6,73	7,05
0,03	2,31	12,42	14,48	14,51
0,23	5,34	7,12	9,06	9,65
0,04	0,06	5,29	7,04	7,28
-0,03	0,37	5,52	8,06	7,12
0,06	0,23	2,49	5,78	7,66
0,09	0,15	3,91	7,71	9,08
0,16	0,08	3,14	4,37	8,44
0,00	-0,09	0,06	2,08	3,95
-0,08	0,00	0,86	1,72	2,61
0,08	0,09	2,53	6,33	6,88
0,06	0,82	9,89	13,12	12,22
0,05	3,12	6,79	7,76	8,03
0,09	0,51	11,74	13,60	13,92
-0,06	0,01	0,76	6,95	9,19
-0,01	2,66	6,10	11,35	11,66
0,06	0,09	10,14	14,15	14,95
0,17	0,36	0,74	4,18	5,47
0,06	1,35	7,20	9,04	10,12
0,06	0,98	8,96	12,32	13,60
0,02	0,23	4,92	13,51	13,82
0,11	1,16	0,86	7,25	7,95
0,03	0,06	5,99	7,69	7,71
-0,32	1,39	8,22	8,79	7,84
0,03	2,06	4,30	7,62	7,09
0,05	0,12	2,44	7,14	7,94
0,58	0,39	4,50	7,96	8,34
0,03	0,11	7,12	8,46	8,26
0,06	0,08	2,68	4,68	5,76
0,03	0,03	0,39	2,11	4,27
0,04	0,01	0,14	5,76	6,19
0,08	0,09	2,64	3,48	6,04
0,58	0,39	4,50	7,96	9,54

0,03	0,11	7,12	8,46	8,26
0,06	0,08	2,68	4,68	4,76
0,03	0,03	0,39	2,11	5,27
0,04	0,01	0,14	5,76	6,19
0,03	0,05	2,99	4,75	4,79
-0,08	0,00	1,99	4,03	4,71
0,11	0,12	2,91	4,54	6,17
0,11	0,43	3,78	9,96	10,72
-0,05	0,08	0,92	7,75	8,93
0,14	0,05	1,42	8,96	12,01
0,05	0,20	4,03	10,48	10,69
-0,02	0,12	2,39	5,76	6,11
0,05	0,14	5,96	8,24	9,21
0,17	-0,09	2,93	3,54	6,46
0,00	7,26	9,45	11,26	10,89
0,09	0,15	4,78	6,74	8,58
