# Supplementary material for: Docosahexaenoic acid, but not eicosapentaenoic acid, improves septic shock-induced arterial dysfunction in rats
Source: PLoS One. 2017 Dec 20;12(12):e0189658. doi: 10.1371/journal.pone.0189658 (PMC5738044; doi:10.1371/journal.pone.0189658)
Supplement: S3 Table — (PDF) [file pone.0189658.s003.pdf]

Table S3

Mesenteric resistance arteries: contraction under Indomethacin

	RAT	Phe 10^{-9}	Phe 3.10^{-9}	Phe 10^{-8}	Phe 3.10^{-8}	Phe 10^{-7}
RAT SHAM G	R1	0,01	-0,05	-0,05	0,00	-0,02
	R2	0,15	0,16	0,20	0,35	0,52
	R3	0,03	0,06	0,08	0,09	0,09
	R4	0,01	-0,31	-0,14	-0,08	-0,09
	R5	-0,02	0,08	0,03	0,06	0,19
	R6	-0,01	0,06	0,14	-0,14	-0,01
	R7	-0,02	-0,03	-0,03	-0,03	0,18
	R8	0,00	0,00	0,00	0,05	0,08
	R9	0,03	-0,02	0,02	0,05	0,03
	R10	-0,03	-0,03	-0,03	0,11	0,02
RAT SHAM EI	R1	0,00	0,02	0,02	0,00	0,00
	R2	0,02	-0,05	-0,09	-0,06	-0,03
	R3	0,00	0,00	0,00	-0,01	-0,01
	R4	0,06	0,11	0,03	0,11	0,50
	R5	-0,06	0,32	0,12	0,29	0,18
	R6	0,04	-0,06	0,04	0,12	0,40
	R7	-0,28	-0,25	-0,17	-0,53	-0,12
	R8	-0,13	-0,18	-0,01	-0,05	-0,02
	R9	-0,02	0,04	-0,09	0,03	-0,22
	R10	0,05	0,05	0,02	0,00	0,06
RAT SHAM D	R1	-0,06	-0,15	-0,28	-0,18	0,04
	R2	0,00	0,00	0,08	0,15	0,54
	R3	0,00	-0,01	-0,03	-0,11	-0,06
	R4	-0,03	0,00	0,02	-0,09	-0,05
	R5	0,09	0,03	0,12	0,09	0,21
	R6	0,06	0,06	0,14	0,35	1,26
	R7	0,03	0,03	0,06	0,14	0,61
	R8	-0,02	-0,03	0,09	0,06	0,34
	R9	0,02	-0,03	0,08	0,18	0,35
RAT SHAM O	R1	-0,15	-0,02	-0,03	-0,08	0,00
	R2	-0,03	-0,03	-0,08	-0,03	-0,02
	R3	0,00	-0,02	0,00	0,06	0,67
	R4	0,02	0,06	0,03	0,05	0,06
	R5	0,06	0,06	0,07	0,06	0,08
	R6	0,34	0,34	0,09	0,26	0,41
	R7	-0,08	0,06	0,11	-0,01	-0,04
	R8	0,26	0,18	0,38	0,34	0,63
RAT SEPSIS G	R1	-0,11	-0,14	0,00	-0,02	-0,03
	R2	-0,14	-0,23	-0,45	-0,22	-0,23
	R3	0,18	0,14	0,14	0,15	0,03
	R4	0,06	0,05	0,12	0,02	0,05

	R5	0,09	0,06	0,08	0,06	0,08
	R6	-0,11	0,00	-0,06	0,04	0,03
	R7	0,00	0,02	-0,04	-0,01	-0,04
	R8	-0,01	-0,01	0,05	-0,01	-0,01
RAT SEPSIS E	R1	0,03	0,03	0,02	0,03	0,00
	R2	0,00	0,00	0,08	0,00	0,02
	R3	0,00	-0,02	0,00	0,03	0,08
	R4	0,14	0,14	0,08	0,06	0,12
	R5	0,05	0,05	0,06	0,06	0,06
	R6	0,03	0,03	-0,05	-0,14	0,09
	R7	0,00	0,05	0,08	0,24	0,03
	R8	0,01	0,04	0,01	-0,05	0,00
	R9	0,00	0,01	0,01	-0,05	0,00
RAT SEPSIS D	R1	0,03	0,02	0,00	-0,02	-0,02
	R2	-0,05	-0,09	-0,12	-0,11	0,55
	R3	0,00	-0,02	0,00	-0,03	0,05
	R4	-0,36	-0,09	-0,12	-0,12	-0,09
	R5	0,02	0,02	0,00	0,02	0,04
	R6	0,11	0,06	0,14	0,16	0,25
	R7	-0,02	0,00	-0,02	-0,02	-0,02
	R8	-0,06	-0,01	0,16	-0,01	0,09
RAT SEPSIS C	R1	0,00	0,00	-0,03	-0,02	-0,03
	R2	0,08	-0,02	0,06	0,02	0,05
	R3	0,08	0,06	0,05	0,05	0,06
	R4	0,00	0,00	0,01	0,00	-0,02
	R5	-0,01	0,03	0,00	0,00	-0,01
	R6	0,00	0,00	0,00	0,05	0,06
	R7	-0,02	-0,02	0,03	0,03	0,04
	R8	-0,05	0,03	-0,06	0,01	0,06

Phe 3.10^{-7}	Phe 10^{-6}	Phe 3.10^{-6}	Phe 10^{-5}	Phe 3.10^{-5}
0,43	5,30	10,35	11,51	11,56
1,73	12,74	14,12	15,98	15,59
9,45	12,29	12,69	12,54	12,50
0,72	6,06	9,85	10,88	10,72
2,29	11,34	12,69	12,20	12,11
0,56	8,34	12,21	12,01	12,16
1,65	8,36	10,31	10,76	11,01
1,06	7,14	12,21	12,16	12,52
0,11	2,57	6,91	7,48	7,63
0,28	3,08	7,31	8,27	8,21
0,06	0,48	12,68	12,85	12,26
-0,03	0,28	13,21	13,38	13,38
5,42	7,95	10,79	11,89	12,23
2,37	6,62	7,06	7,36	7,49
1,36	2,46	11,06	12,01	12,35
2,59	6,68	8,26	10,44	10,32
0,55	2,04	6,46	9,71	9,88
0,45	3,09	6,07	9,24	9,51
0,98	4,87	9,32	10,20	10,55
0,55	5,96	10,16	10,01	10,74
0,23	3,46	12,03	13,79	12,73
3,89	7,02	7,74	8,04	8,26
1,12	3,16	11,51	11,51	11,89
-0,06	7,26	11,05	11,72	12,26
3,06	8,21	10,86	11,42	11,59
4,67	10,31	12,47	12,41	12,41
1,90	9,38	12,02	12,96	12,80
1,02	12,35	12,72	12,05	12,71
2,42	7,23	11,66	11,78	11,81
0,71	11,23	11,74	12,73	12,52
0,18	8,55	12,52	13,59	13,45
1,81	6,98	9,04	9,62	9,44
0,09	2,79	6,49	10,86	10,83
0,45	5,62	12,04	12,93	13,35
1,68	5,79	8,16	9,93	9,98
2,76	3,48	7,56	8,76	8,88
2,15	5,86	10,34	11,69	11,91
0,14	0,35	5,21	7,00	7,54
4,34	5,61	9,41	9,77	9,82
-0,01	0,17	2,93	8,89	8,91
3,34	5,34	6,21	7,31	7,75

0,08	2,31	4,56	7,71	7,50
0,11	2,37	9,18	9,42	9,46
0,12	5,34	8,41	9,41	9,28
0,48	3,92	10,32	11,11	9,65
0,02	0,03	1,24	7,40	7,80
0,15	3,11	7,49	7,89	7,89
0,05	0,76	6,59	9,29	9,52
0,15	0,32	6,85	9,04	9,58
0,34	1,87	7,02	7,36	7,49
4,08	6,63	7,99	8,20	8,59
0,52	4,34	8,43	9,16	9,55
3,66	8,65	8,76	9,83	10,09
0,11	0,11	6,11	8,43	8,40
0,00	0,14	1,52	1,98	4,23
2,82	10,89	12,65	12,06	12,02
0,26	0,96	7,96	9,18	9,45
0,00	0,12	3,52	4,75	5,07
0,06	0,34	5,07	8,46	9,53
0,59	7,60	11,00	11,75	11,94
0,05	4,87	11,43	12,98	12,49
4,25	4,74	9,95	9,61	9,69
0,01	1,38	7,52	8,16	8,41
0,12	5,38	8,67	11,39	11,73
0,19	0,58	5,72	7,56	7,73
0,24	0,11	6,91	9,26	8,75
1,18	5,33	7,23	7,81	8,63
-0,03	0,59	5,87	7,44	7,73
1,30	7,46	8,18	8,86	9,08
0,23	0,24	7,16	12,15	13,16
