# Supplementary material for: Docosahexaenoic acid, but not eicosapentaenoic acid, improves septic shock-induced arterial dysfunction in rats
Source: PLoS One. 2017 Dec 20;12(12):e0189658. doi: 10.1371/journal.pone.0189658 (PMC5738044; doi:10.1371/journal.pone.0189658)
Supplement: S4 Table — (PDF) [file pone.0189658.s004.pdf]

Table S4

Mesenteric resistance arteries: contraction under association of L-NA & Indomethacin

	RAT	Phe 10^{-9}	Phe 3.10^{-9}	Phe 10^{-8}	Phe 3.10^{-8}	Phe 10^{-7}
	R1	0,01	-0,05	-0,05	0,00	-0,02
	R2	0,15	0,16	0,20	0,35	0,52
	R3	0,03	0,06	0,08	0,09	0,09
	R4	0,01	-0,31	-0,14	-0,08	-0,09
**RAT SHAM G5**	R5	-0,02	0,08	0,03	0,06	0,19
	R6	-0,01	0,06	0,14	-0,14	-0,01
	R7	-0,02	-0,03	-0,03	-0,03	0,18
	R8	0,00	0,00	0,00	0,05	0,08
	R9	0,03	-0,02	0,02	0,05	0,03
	R10	-0,03	-0,03	-0,03	0,11	0,02
	R1	0,00	0,02	0,02	0,00	0,00
	R2	0,02	-0,05	-0,09	-0,06	-0,03
	R3	0,00	0,00	0,00	-0,01	-0,01
	R4	0,06	0,11	0,03	0,11	0,50
**RAT SHAM EPA**	R5	-0,06	0,32	0,12	0,29	0,18
	R6	0,04	-0,06	0,04	0,12	0,40
	R7	-0,28	-0,25	-0,17	-0,53	-0,12
	R8	-0,13	-0,18	-0,01	-0,05	-0,02
	R9	-0,02	0,04	-0,09	0,03	-0,22
	R10	0,05	0,05	0,02	0,00	0,06
	R1	-0,06	-0,15	-0,28	-0,18	0,04
	R2	0,00	0,00	0,08	0,15	0,54
	R3	0,00	-0,01	-0,03	-0,11	-0,06
	R4	-0,03	0,00	0,02	-0,09	-0,05
**RAT SHAM DHA**	R5	0,09	0,03	0,12	0,09	0,21
	R6	0,06	0,06	0,14	0,35	1,26
	R7	0,03	0,03	0,06	0,14	0,61
	R8	-0,02	-0,03	0,09	0,06	0,34
	R9	0,02	-0,03	0,08	0,18	0,35
	R1	-0,15	-0,02	-0,03	-0,08	0,00
	R2	-0,03	-0,03	-0,08	-0,03	-0,02
	R3	0,00	-0,02	0,00	0,06	0,67
**RAT SHAM OM**	R4	0,02	0,06	0,03	0,05	0,06
	R5	0,06	0,06	0,07	0,06	0,08
	R6	0,34	0,34	0,09	0,26	0,41
	R7	-0,08	0,06	0,11	-0,01	-0,04
	R8	0,26	0,18	0,38	0,34	0,63
	R1	-0,11	-0,14	0,00	-0,02	-0,03
	R2	-0,14	-0,23	-0,45	-0,22	-0,23
	R3	0,18	0,14	0,14	0,15	0,03
**RAT SEPSIS**	R4	0,06	0,05	0,12	0,02	0,05

**G5**	R5	0,09	0,06	0,08	0,06	0,08
	R6	-0,11	0,00	-0,06	0,04	0,03
	R7	0,00	0,02	-0,04	-0,01	-0,04
	R8	-0,01	-0,01	0,05	-0,01	-0,01
	R1	0,03	0,03	0,02	0,03	0,00
	R2	0,00	0,00	0,08	0,00	0,02
	R3	0,00	-0,02	0,00	0,03	0,08
	R4	0,14	0,14	0,08	0,06	0,12
**RAT SEPSIS EPA**	R5	0,05	0,05	0,06	0,06	0,06
	R6	0,03	0,03	-0,05	-0,14	0,09
	R7	0,00	0,05	0,08	0,24	0,03
	R8	0,01	0,04	0,01	-0,05	0,00
	R9	0,00	0,01	0,01	-0,05	0,00
	R1	0,03	0,02	0,00	-0,02	-0,02
	R2	-0,05	-0,09	-0,12	-0,11	0,55
	R3	0,00	-0,02	0,00	-0,03	0,05
**RAT SEPSIS DHA**	R4	-0,36	-0,09	-0,12	-0,12	-0,09
	R5	0,02	0,02	0,00	0,02	0,04
	R6	0,11	0,06	0,14	0,16	0,25
	R7	-0,02	0,00	-0,02	-0,02	-0,02
	R8	-0,06	-0,01	0,16	-0,01	0,09
	R1	0,00	0,00	-0,03	-0,02	-0,03
	R2	0,08	-0,02	0,06	0,02	0,05
	R3	0,08	0,06	0,05	0,05	0,06
**RAT SEPSIS OM**	R4	0,00	0,00	0,01	0,00	-0,02
	R5	-0,01	0,03	0,00	0,00	-0,01
	R6	0,00	0,00	0,00	0,05	0,06
	R7	-0,02	-0,02	0,03	0,03	0,04
	R8	-0,05	0,03	-0,06	0,01	0,06

Phe 3.10^{-7}	Phe 10^{-6}	Phe 3.10^{-6}	Phe 10^{-5}	Phe 3.10^{-5}
0,43	5,30	10,35	11,51	11,56
1,73	9,74	10,12	11,98	11,59
9,45	12,29	12,69	12,54	12,50
0,72	6,06	9,85	10,88	10,72
2,29	11,34	11,69	11,20	11,11
0,56	8,34	12,21	12,01	12,16
1,65	8,36	10,31	10,76	11,01
1,06	7,14	12,21	12,16	12,52
0,11	2,57	4,91	5,48	5,63
0,28	3,08	7,31	8,27	8,21
0,06	0,48	12,68	12,85	12,26
-0,03	0,28	13,21	13,12	13,12
5,42	7,95	10,79	11,89	11,23
2,37	6,62	7,06	7,36	7,49
1,36	2,46	11,06	12,01	12,35
2,59	6,68	8,26	10,44	10,32
0,55	2,04	6,46	9,40	9,20
0,45	3,09	6,07	6,24	6,51
0,98	4,87	9,32	10,20	10,55
0,55	5,96	10,16	10,01	10,05
0,23	3,46	12,03	16,00	16,02
3,89	7,02	7,74	8,04	8,10
1,12	3,16	11,51	11,51	11,89
-0,06	7,26	11,05	11,72	12,26
3,06	8,21	10,86	11,42	11,59
4,67	10,31	12,47	12,41	12,41
1,90	9,38	13,02	16,96	16,80
1,02	6,35	6,72	7,05	7,71
2,42	7,23	11,66	11,78	11,81
0,71	11,23	11,74	12,73	12,52
0,18	8,55	12,52	13,59	13,45
1,81	6,98	9,04	9,62	9,44
0,09	2,79	6,49	9,86	9,83
0,45	5,62	12,04	14,93	14,35
1,68	5,79	8,16	9,93	9,98
2,76	3,48	7,56	8,76	8,88
2,15	5,86	10,34	12,69	12,91
0,14	0,35	5,21	7,00	7,54
4,34	5,61	10,41	14,77	14,82
-0,01	0,17	2,93	8,89	8,91
3,34	5,34	6,21	7,31	7,75

0,08	2,31	4,56	7,71	7,80
0,11	2,37	9,18	9,42	9,46
0,12	5,34	8,41	9,41	9,28
0,48	3,92	10,32	11,11	9,65
0,02	0,03	1,24	5,40	5,80
0,15	3,11	7,49	7,89	7,89
0,05	0,76	6,59	9,29	9,52
0,15	0,32	6,85	8,45	8,58
0,34	1,87	7,02	8,36	8,49
4,08	6,63	7,99	8,20	8,59
0,52	4,34	8,43	9,16	9,55
3,66	8,65	8,76	9,83	10,09
0,11	0,11	6,11	8,43	8,40
0,00	0,14	2,52	7,98	7,23
2,82	10,89	10,65	10,06	10,02
0,26	0,96	7,96	9,18	9,45
0,00	0,12	3,52	4,75	5,07
0,06	0,34	5,07	8,46	9,53
0,59	7,60	11,00	11,75	11,94
0,05	4,87	8,43	8,98	8,49
4,25	4,74	9,95	9,61	9,69
0,01	1,38	7,52	8,16	8,41
0,12	5,38	8,67	11,39	11,73
0,19	0,58	6,72	9,56	9,73
0,24	0,11	6,91	9,26	8,75
1,18	5,33	7,23	10,81	10,63
-0,03	0,59	5,87	8,44	8,73
1,30	7,46	8,18	9,86	9,08
0,23	0,24	10,16	15,15	15,16
