# Supplementary material for: Docosahexaenoic acid, but not eicosapentaenoic acid, improves septic shock-induced arterial dysfunction in rats
Source: PLoS One. 2017 Dec 20;12(12):e0189658. doi: 10.1371/journal.pone.0189658 (PMC5738044; doi:10.1371/journal.pone.0189658)
Supplement: S5 Table — (PDF) [file pone.0189658.s005.pdf]

Table S5

Mesenteric resistance arteries: relaxation to Ach without inhibitor

	RAT	Ach 10^{-10}	Ach 3.10^{-10}	Ach 10^{-9}	Ach 3.10^{-9}	Ach 10^{-8}
**RAT SHAM G5**	R1	100,00%	98,68%	96,14%	75,46%	1,31%
	R2	100,00%	71,52%	40,04%	23,43%	5,26%
	R3	100,00%	69,31%	31,65%	15,76%	8,18%
	R4	100,00%	88,64%	53,41%	32,95%	44,43%
	R5	100,00%	98,87%	96,09%	91,17%	20,37%
	R6	100,00%	81,88%	74,08%	64,51%	55,87%
**RAT SEPSIS G5**	R1	100,00%	100,98%	94,51%	89,74%	82,75%
	R2	100,00%	85,19%	81,85%	73,59%	63,73%
	R3	100,00%	99,32%	80,77%	74,63%	54,30%
	R4	100,00%	96,87%	95,78%	91,69%	55,93%
	R5	100,00%	75,54%	67,03%	59,96%	51,99%
	R6	100,00%	106,62%	108,35%	108,04%	101,42%
	R7	100,00%	101,54%	100,81%	91,26%	75,48%
	R8	100,00%	94,41%	88,83%	85,60%	77,01%
	R9	100,00%	84,13%	67,04%	68,83%	62,16%
	R10	100,00%	83,93%	79,14%	69,94%	69,94%
**RAT SEPSIS EP**	R1	100,00%	96,58%	89,34%	57,04%	40,04%
	R2	100,00%	90,49%	81,64%	78,36%	49,74%
	R3	100,00%	99,44%	101,16%	106,93%	96,35%
	R4	100,00%	57,40%	34,74%	33,38%	49,31%
	R5	100,00%	111,62%	100,62%	86,10%	41,29%
	R6	100,00%	81,67%	75,26%	70,65%	67,49%
	R7	100,00%	67,86%	43,86%	40,19%	41,14%
	R8	100,00%	95,17%	100,00%	100,71%	60,36%
**RAT SEPSIS DI**	R1	100,00%	100,38%	95,27%	82,19%	72,05%
	R2	100,00%	90,53%	81,58%	66,39%	53,64%
	R3	100,00%	70,77%	55,58%	51,31%	50,41%
	R4	100,00%	90,42%	69,95%	34,88%	6,23%
	R5	100,00%	81,54%	73,84%	59,81%	28,27%
	R6	100,00%	91,55%	88,08%	84,13%	22,73%
	R7	100,00%	90,86%	80,99%	80,27%	74,33%
	R8	100,00%	97,89%	95,96%	95,82%	93,89%
	R9	100,00%	89,52%	78,91%	73,76%	67,57%
**RAT SEPSIS OI**	R1	100,00%	89,41%	82,49%	75,87%	39,79%
	R2	100,00%	84,38%	93,68%	76,53%	64,07%
	R3	100,00%	62,97%	40,02%	25,65%	17,38%
	R4	100,00%	69,86%	58,70%	26,47%	8,12%
	R5	100,00%	84,54%	69,74%	50,00%	32,57%
	R6	100,00%	100,00%	74,62%	98,07%	47,06%
	R7	100,00%	93,97%	92,72%	63,20%	47,25%
	R8	100,00%	85,44%	84,55%	74,71%	64,35%

Ach 3.10^{-8}	Ach 10^{-7}	Ach 3.10^{-7}	Ach 10^{-6}	Ach 3.10^{-6}	Ach 10^{-5}	Ach 3.10^{-5}
1,27%	4,69%	5,91%	0,11%	0,11%	0,00%	0,11%
4,91%	1,93%	0,83%	-1,03%	-1,03%	-0,83%	-1,03%
9,82%	8,39%	2,45%	0,20%	0,20%	1,64%	0,00%
26,14%	21,25%	21,25%	5,80%	5,80%	3,86%	2,27%
1,93%	1,18%	0,96%	0,29%	0,00%	0,00%	0,13%
18,04%	12,77%	22,19%	0,32%	0,32%	0,00%	0,19%
66,73%	54,84%	38,30%	33,27%	15,49%	14,33%	14,64%
49,02%	40,00%	23,52%	22,68%	7,94%	7,34%	2,02%
44,90%	43,37%	27,46%	21,20%	10,75%	10,34%	0,00%
40,05%	35,97%	-0,75%	0,00%	-0,54%	0,00%	-0,75%
39,58%	41,12%	26,64%	23,56%	11,23%	9,69%	-0,18%
98,89%	58,50%	35,06%	24,18%	12,62%	11,56%	-0,49%
5,58%	1,17%	1,32%	0,95%	0,81%	0,81%	0,37%
64,18%	57,88%	45,70%	33,85%	15,33%	14,13%	2,79%
13,24%	1,97%	6,85%	5,27%	0,00%	1,28%	1,22%
65,77%	72,88%	68,22%	64,66%	25,24%	23,15%	2,21%
34,21%	11,86%	-0,39%	-1,45%	0,00%	-1,45%	-2,11%
2,34%	2,43%	0,00%	2,21%	0,00%	0,26%	0,00%
12,90%	3,21%	0,55%	0,65%	3,16%	-0,13%	-0,64%
24,65%	5,74%	10,79%	0,00%	6,16%	5,74%	7,25%
40,66%	2,70%	2,47%	3,94%	0,00%	6,02%	1,24%
44,32%	31,61%	5,13%	5,30%	1,31%	2,31%	1,81%
44,45%	9,93%	-1,39%	0,00%	-2,50%	-2,50%	-0,97%
51,60%	-1,15%	-0,44%	0,00%	0,53%	-1,33%	0,00%
14,27%	4,88%	1,58%	1,20%	-0,38%	-0,15%	-0,60%
15,04%	6,76%	0,42%	-0,26%	0,05%	-0,83%	-0,16%
41,22%	6,90%	0,00%	-1,97%	-1,72%	-1,97%	-1,97%
0,19%	0,47%	3,53%	0,19%	-0,56%	1,40%	1,21%
30,49%	8,23%	2,95%	1,05%	0,74%	0,74%	0,74%
9,98%	2,36%	1,32%	1,80%	1,11%	1,66%	1,32%
20,77%	3,72%	1,05%	-0,20%	0,72%	0,20%	0,52%
91,92%	63,91%	7,71%	1,46%	0,99%	0,61%	0,85%
59,41%	6,87%	0,37%	1,29%	1,29%	1,47%	2,27%
13,44%	7,85%	11,12%	3,48%	1,19%	16,19%	1,04%
33,98%	7,03%	1,88%	0,91%	0,91%	0,00%	0,12%
5,90%	0,98%	1,06%	0,00%	-0,19%	0,00%	-0,48%
6,94%	2,29%	2,03%	-1,59%	-2,39%	-2,89%	-2,39%
14,12%	5,10%	1,96%	0,00%	-0,66%	-0,41%	-0,66%
1,20%	1,20%	1,77%	0,00%	-1,04%	-1,04%	-1,04%
13,80%	1,85%	1,62%	0,60%	0,48%	-0,18%	0,48%
24,29%	2,20%	0,50%	0,50%	0,40%	0,65%	1,04%

Ach 10^{-4}
0,55%
-1,65%
0,00%
0,00%
0,00%
0,94%
14,05%
1,01%
0,00%
-1,77%
-0,45%
-0,68%
0,37%
2,79%
1,03%
2,45%
-0,39%
-1,69%
0,00%
4,83%
3,94%
3,42%
-0,97%
-0,18%
-0,60%
-1,35%
-1,97%
-3,44%
0,74%
1,11%
0,33%
0,52%
1,29%
1,19%
0,12%
-0,19%
-3,88%
0,16%
-0,88%
0,00%
1,04%
