# Supplementary material for: Docosahexaenoic acid, but not eicosapentaenoic acid, improves septic shock-induced arterial dysfunction in rats
Source: PLoS One. 2017 Dec 20;12(12):e0189658. doi: 10.1371/journal.pone.0189658 (PMC5738044; doi:10.1371/journal.pone.0189658)
Supplement: S6 Table — (PDF) [file pone.0189658.s006.pdf]

Table S6

WB

MRA iNOS	SHAM-D5	CLP-D5	CLP-EPA	CLP-DHA	CLP-EPA/DHA
	0,056	0,309	0,186	0,176	0,151
	0,131	0,294	0,189	0,181	0,111
	0,097	0,274	0,271	0,071	0,037
		0,168	0,219	0,051	0,141
				0,19	0,195
				0,165	0,098

MRA piKB	SHAM-D5	CLP-D5	CLP-EPA	CLP-DHA	CLP-EPA/DHA
	0,086	0,119	0,078	0,081	0,081
	0,031	0,137	0,102	0,123	0,075
	0,065	0,147	0,155	0,125	0,079
	0,042	0,179	0,129	0,038	0,089
	0,087		0,154	0,045	0,077
	0,152		0,141		0,059

MRA NfkB	SHAM-D5	CLP-D5	CLP-EPA	CLP-DHA	CLP-EPA/DHA
	0,172	0,464	0,31	0,311	0,171
	0,324	0,776	0,469	0,253	0,333
	0,313	0,522	0,427	0,282	0,332
	0,217		0,324	0,373	0,182
	0,224		0,39		0,252
	0,136				

MRA COX2	SHAM-D5	CLP-D5	CLP-EPA	CLP-DHA	CLP-EPA/DHA
	0,053	0,411	0,384	0,247	0,182
	0,249	0,322	0,691	0,189	0,146
	0,121	0,311	0,337	0,212	0,151
	0,161	0,336	0,433	0,241	0,097
					0,223

MRA HO1	SHAM-D5	CLP-D5	CLP-EPA	CLP-DHA	CLP-EPA/DHA
	0,158	0,115	0,234	0,268	0,368
	0,124	0,196	0,178	0,398	0,551
	0,123	0,102	0,16	0,397	0,4
	0,165	0,157	0,259	0,447	0,345

0,489	0,252	0,184	0,328	0,346
