# Supplementary material for: Docosahexaenoic acid, but not eicosapentaenoic acid, improves septic shock-induced arterial dysfunction in rats
Source: PLoS One. 2017 Dec 20;12(12):e0189658. doi: 10.1371/journal.pone.0189658 (PMC5738044; doi:10.1371/journal.pone.0189658)
Supplement: S7 Table — (PDF) [file pone.0189658.s007.pdf]

Table s7

EPR

NO aorta	SHAM-D5	CLP-D5	CLP-EPA	CLP-DHA	CLP-EPA/DHA
	271,7	435,52	483,75	409	331,08
	234,29	500,86	516,39	347,06	418,46
	232,7	464,05	427,59	411,25	399,5
	258,85	412,75	452,5	417,83	409,43
	389,15	511,7	711,67	417,69	373,95
	378,84	505,1	619,56	440,34	316,74
	385,13	402,6	520,7		326,59
	235,76	567,62	566,15		352,08
	318,29	691,67			

02 aorta	SHAM-D5	CLP-D5	CLP-EPA	CLP-DHA	CLP-EPA/DHA
	781,01	2161,05	2344,58	1032,92	704,26
	677,06	1726,76	1211,27	1425,14	706,56
	720,94	1924,35	1629,84	1020,23	1033,11
	523,33	1798,28	2406,52	946,17	1226,84
	650,06	2124,69	2285,24	1136,07	1350,12
	840,65	1323,58	1815,65	1691,19	1441
	741,48	1610,74	1805,21	1382,63	1125,35
	991,32	1485,5	1847,19	887,73	1314,33
	940,19	1357,26	2626,79	1358,89	1452,89
	971,55			1434	1102,42
				1218,65	
				1033,09	
