# Supplementary material for: Docosahexaenoic acid, but not eicosapentaenoic acid, improves septic shock-induced arterial dysfunction in rats
Source: PLoS One. 2017 Dec 20;12(12):e0189658. doi: 10.1371/journal.pone.0189658 (PMC5738044; doi:10.1371/journal.pone.0189658)
Supplement: S8 Table — (PDF) [file pone.0189658.s008.pdf]

Table S8

TXB2	SHAM-D5	CLP-D5	CLP-EPA	CLP-DHA	CLP-EPA/DHA
	1,58	1,65	1,08	1,88	0,94
	1,15	1,82	0,89	1,25	1,99
	1,71	3,9	1,44	2,76	1,84
	1,62	2,21	1,34	1,34	2,24

keto	SHAM-D5	CLP-D5	CLP-EPA	CLP-DHA	CLP-EPA/DHA
	0,2	0,83	0,47	0,69	0,53
	0,26	0,91	0,73	0,4	0,5
	0,24	0,46	1,69	0,64	0,39
	0,27	1,85	1,56	0,46	0,39
		0,39	2,78	0,33	
