# Supplementary figures and images for: Docosahexaenoic acid, but not eicosapentaenoic acid, improves septic shock-induced arterial dysfunction in rats
Source: PLoS One. 2017 Dec 20;12(12):e0189658. doi: 10.1371/journal.pone.0189658 (PMC5738044; doi:10.1371/journal.pone.0189658)

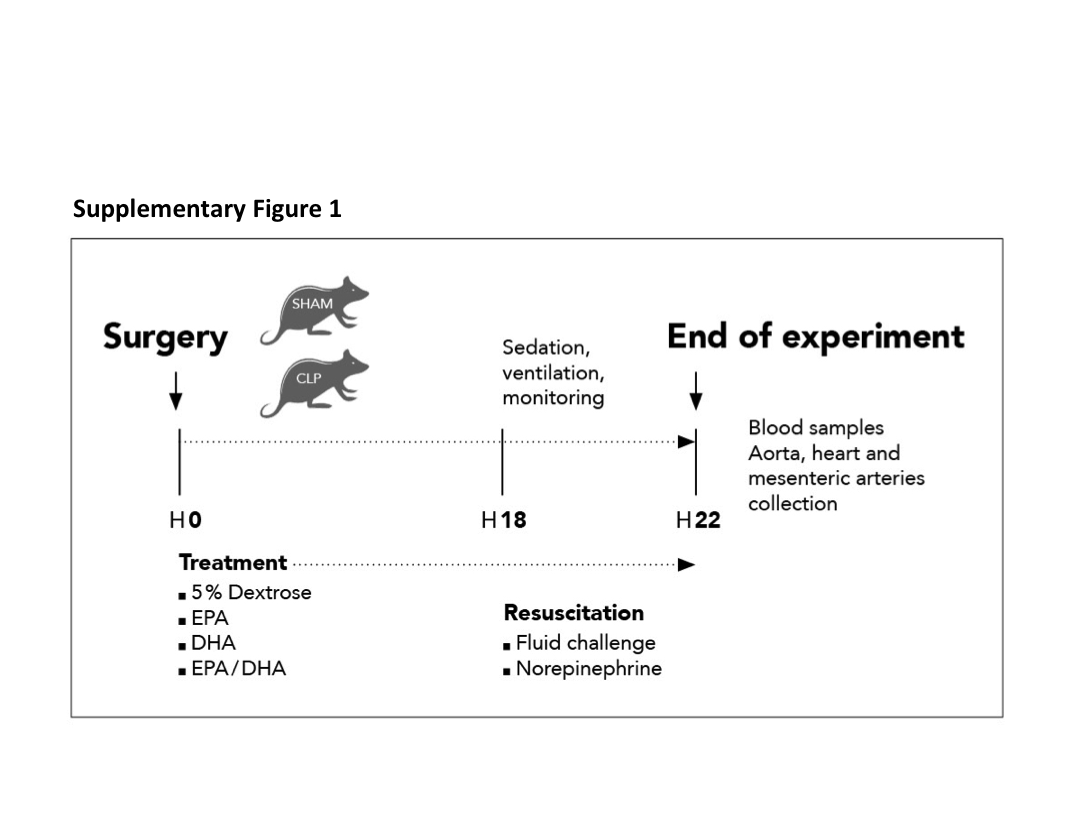

Supplement: S1 Fig — At H0, rats were randomly allocated to a group and underwent a surgical procedure (either cecal ligation and puncture, CLP, or SHAM) and were infused until H22 by 5% dextrose (D5), purified EPA, DHA or a mixture of EPA and DHA at identical rates. From H18 to H22, rats were anesthetized, ventilated and resuscitated with fluid challenge and norepinephrine to reach the mean arterial pressure (MAP) target over 90 mmHg. At H22, rats were sacrificed. n = 10 rats/group. (TIF) [file pone.0189658.s009.tif]
